# Construct Validity of a Serious Game for Laparoscopic Skills Training: Validation Study

**DOI:** 10.2196/17222

**Published:** 2020-05-07

**Authors:** Wouter IJgosse, Harry van Goor, Camiel Rosman, Jan-Maarten Luursema

**Affiliations:** 1 Department of Surgery Radboud University Medical Center Nijmegen Netherlands

**Keywords:** laparoscopy, surgery, training, education, serious game, resident training, skills development, psychomotor skills, simulation center

## Abstract

**Background:**

Surgical residents underutilize opportunities for traditional laparoscopic simulation training. Serious gaming may increase residents’ motivation to practice laparoscopic skills. However, little is known about the effectiveness of serious gaming for laparoscopic skills training.

**Objective:**

The aim of this study was to establish construct validity for the laparoscopic serious game *Underground*.

**Methods:**

All study participants completed 2 levels of *Underground*. Performance for 2 novel variables (time and error) was compared between novices (n=65, prior experience <10 laparoscopic procedures), intermediates (n=26, prior experience 10-100 laparoscopic procedures), and experts (n=20, prior experience >100 laparoscopic procedures) using analysis of covariance. We corrected for gender and video game experience.

**Results:**

Controlling for gender and video game experience, the effects of prior laparoscopic experience on the time variable differed significantly (*F*_2,106_=4.77, *P*=.01). Both experts and intermediates outperformed novices in terms of task completion speed; experts did not outperform intermediates. A similar trend was seen for the rate of gameplay errors. Both gender (*F*_1,106_=14.42, *P*<.001 in favor of men) and prior video game experience (*F*_1,106_=5.20, *P*=.03 in favor of experienced gamers) modulated the time variable.

**Conclusions:**

We established construct validity for the laparoscopic serious game *Underground*. Serious gaming may aid laparoscopic skills development. Previous gaming experience and gender also influenced *Underground* performance. The in-game performance metrics were not suitable for statistical evaluation. To unlock the full potential of serious gaming for training, a more formal approach to performance metric development is needed.

## Introduction

Simulation has been proven to be effective for laparoscopic skills training [1]. However, due to scheduling constraints and motivational issues, simulation training opportunities for residents remain underutilized [2,3]. Consequently, the burden of the surgical learning curve continues to fall on the patient [4-6]. Serious gaming can address these issues by providing a training modality that is fun, challenging, easy to implement, and inexpensive compared to current laparoscopic simulators [7]. Serious gaming refers to the application or adaptation of computer games for nonrecreational purposes, such as learning, training, or therapy [8]. This can lead to “stealth learning” [9], where the trainee is enjoying the training so much that they fail to notice improvements in key education outcomes [10,11]. Due to these attributes, serious gaming is a good candidate to alleviate motivational issues. Serious games are also easy to implement in a home environment, which can address the issue of scheduling constraints at work.

Serious gaming for psychomotor skills training is a new concept in surgery; however, it has been demonstrated to be successful in several other fields, including rehabilitation clinics and the aviation industry [12-15]. In these games, movements necessary to engage in gameplay are modeled on relevant motor tasks as they occur in reality in order to impart the requisite motor skills. This approach was adopted for laparoscopic skills training by ten Cate Hoedemaker and Grendel Games [16], who developed the serious game *Underground* for the Nintendo Wii U platform. In this game, medical content is replaced by a narrative that focuses on saving robots from a system of abandoned mineshafts. The psychomotor skills necessary to complete in-game tasks are closely modeled on laparoscopic movements and are performed using gaming controllers that resemble laparoscopic hardware. If effective, serious gaming can contribute to laparoscopic skills development by increasing training volume in the guise of a leisure activity.

In a previous study, we established concurrent validity by demonstrating skill transfer between *Underground* and the LapSim virtual reality trainer [17,18]. Limited evidence of construct validity has been offered by Jalink et al [18], who compared the performance of laparoscopic experts and internists playing *Underground*. However, this study compared groups who may differ in their innate abilities to perform psychomotor tasks [19]; also, it used a prototype of the game instead of the final product.

To assess the potential of *Underground* as a surgical training tool, validity criteria must be met, including construct validity [20]. Construct validity is the degree to which a test truly measures what it intends to measure; in this instance, it refers to the degree to which a serious game measures differences in the skills it is designed to evaluate. Therefore, the aim of this study was to establish construct validity for the laparoscopic serious game *Underground*. We compared the gaming performance of surgical novices, surgical house officers, surgical residents, and laparoscopic surgeons based on their self-reported laparoscopic experience. We hypothesized that more real-world laparoscopic experience would translate to better *Underground* gaming performance.

## Methods

### Participants

Study participants were selected from 4 different groups: fourth year medical students preparing for their surgical internships (surgical novices), surgical house officers, surgical residents, and staff laparoscopic surgeons from the departments of Colorectal and Hepatobiliary Surgery, Urology, and Gynecology. All participants were recruited at the Radboud University Medical Center, Nijmegen, the Netherlands. No IRB approval was needed for this study under Dutch law [21].

Performance data for the surgical novices were collected during a mandatory basic laparoscopic skills training course. The participants were informed about our study, and each participant voluntarily signed an informed consent form to allow us to use their anonymized performance data for research purposes. Participants were made aware that declining to sign this form did not impact their course participation or the assessment of their internship. Surgical house officers, surgical residents, and staff surgeons were recruited via emails, posters in the hospital, and in-person interactions. Participation was voluntary and did not result in compensation. All subjects were divided into 3 groups based on their self-reported laparoscopic experience: novices, who had performed fewer than 10 laparoscopic procedures (typically surgical novices and surgical house officers), intermediates, who had performed between 10 and 100 laparoscopic procedures (typically surgical residents), and experts, who had performed more than 100 laparoscopic procedures (typically laparoscopic surgeons) [22].

### Study Design

An instructor was present throughout the session to observe the participants, provide instructions, and troubleshoot the game when necessary. After completing the informed consent form, the participants played the first 2 levels of the laparoscopic serious game *Underground* to familiarize themselves with the software and hardware of the system. Next, they played through the fourth and fifth levels of *Underground* while being timed and scored for error by the instructor. The fourth and fifth levels were selected as the basis of the assessment because of the more challenging nature of these levels, in which all basic laparoscopic skills were tested (eg, inverted movements, hand-eye coordination, depth perception, and ambidexterity). Participants subsequently completed a demographic questionnaire that included sections on prior laparoscopic experience and prior video game experience. Each complete session took an average of 30 minutes per individual.

**Figure 1:**
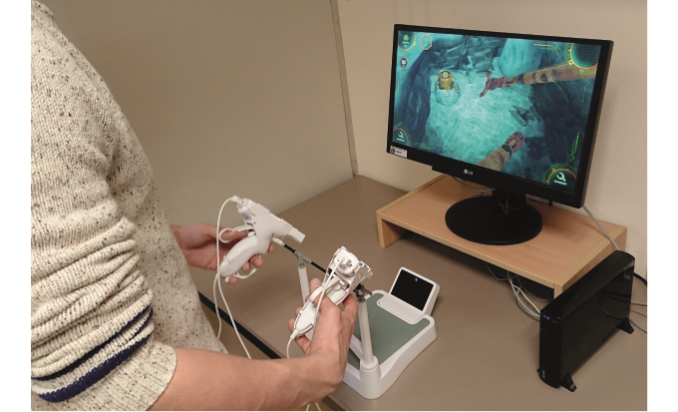
Laparoscopic interface of the serious game *Underground*.

Gaming sessions took place in a quiet working space located within the Department of Surgery (Figure 1). Fluorescent lights and sources of infrared light were turned off in the gaming area, as they are known to interfere with the Nintendo Wii-U system used in this study [23].

### Apparatus

We utilized *Underground* Version 1.1 with a dedicated laparoscopic interface for this study (Grendel Games). The serious game was played on a Nintendo Wii U video gaming system connected to a 21” HD LCD screen (LG Corporation). The game instrument sensitivity was set at maximum [24,25].

### Data Preparation

Currently, *Underground* contains no formal embedded performance metrics relevant to laparoscopic skills training. Thus, in order to analyze game performance as it relates to surgical activities, we created 2 measured variables: time and error. Time was the measurement of how quickly the player was able to complete all game tasks; it was defined as the elapsed time between the start and finish of each level as measured with a stopwatch. The total play times for each level were summed to generate the final time measurement. Error was defined as the total sum of occurrences of 3 key player mistakes: orb drops, roadblocks, and robot deaths.

Video game experience was measured by asking all participants to estimate their average number of weekly gaming hours across several age-bands (1-6 years, 7-12 years, 13-18 years, 19-25 years, 26-45 years, and ≥46 years) [26]. We then calculated each participant’s total prior lifetime gaming experience by multiplying the average number of gaming hours per week by the number of weeks in the selected age bands. Since the resulting total gaming hours variable was not normally distributed, we ranked these data to render them suitable for non-parametric statistical analyses.

### Data Analysis

Sample size was calculated using α=.05, a power of .95, a large effect size of 0.40, and 3 groups [18,27]. This resulted in a desired total sample size of 102. Normality for the variable time was confirmed using the Shapiro-Wilk test, allowing for parametric testing. We performed analysis of covariance (ANCOVA) to assess the effects of laparoscopic experience on the “time” performance variable, controlling for prior video game experience and gender. After performing ANCOVA on all groups together, planned contrasts were used to assess differences in performance for paired groups [28]. The incidence of error was not normally distributed for the novice or expert groups, as assessed by the Shapiro-Wilk test. We therefore used Mann-Whitney U tests to compare the number of errors between groups. α was set at .05. Power analyses were conducted in G*Power version 3.1.9.2. Statistical analyses were performed with SPSS version 25.0 (IBM Corporation).

## Results

### Participants

A total of 120 participants were enrolled in this study. Data for 9 participants (2 surgical novices, 4 surgical residents, 1 surgical house officer, and 2 laparoscopic surgeons) were excluded from the analyses because these participants were unable to complete the study due to hardware failure or urgent patient care activities. Data for 65 novice, 26 intermediate, and 20 expert participants were used. Gender proportions varied between experience groups (Table 1). Men had more video game experience than women in the novice group (*t*_63_=4.94, *P*<.001) and intermediate group (*t*_24_=3.00, *P*=.01). No gender-based difference for video game experience was found in the expert group.

**Table 1:** Demographics of the participant groups.

Characteristic		Novice (n=65)	Intermediate (n=26)	Expert (n=20)
Age (years), mean (SD)		24 (3)	31 (2)	44 (6)
Male gender, n (%)		20 (31)	16 (62)	17 (85)
Right-hand dominance, n (%)		57 (87)	23 (89)	20 (100)
Video game experience, mean rank		56	60	50
**Professional status, n (%)**				
	Surgical novice	54 (83)	N/A^a^	N/A
	Surgical house officer	11 (17)	N/A	N/A
	Surgical resident	N/A	26 (100)	N/A
	Laparoscopic surgeon	N/A	N/A	20 (100)
**Department, n (%)**				
	No department (surgical novice)	54 (83)	N/A	N/A
	Surgery	11 (17)	18 (69)	14 (70)
	Urology	N/A	3 (12)	2 (10)
	Gynecology	N/A	5 (19)	4 (20)

^a^Not applicable.

### Time Performance Variable

**Figure 2:**
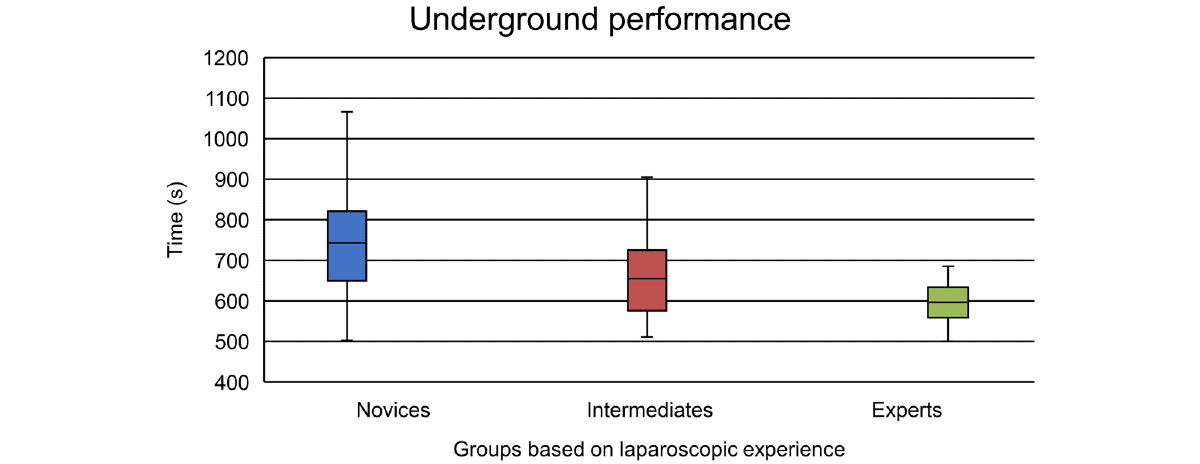
Box plots showing the performance distribution for time between groups. The assessment time is the sum of the time scores for levels 4 and 5 of the serious game *Underground*.

After controlling for gender and prior video game experience by including them as covariates in ANCOVA, the participants’ game completion times differed significantly depending on their laparoscopic experience (*F*_2,106_=4.77, *P*=.01). Planned contrasts revealed that experts (mean difference –88 seconds, 95% CI –146 to –30, *P*=.002) and intermediates (mean difference –43 seconds, 95% CI –92 to 5, *P*=.04) performed faster than novices. Experts did not outperform intermediates (mean difference –45 seconds, 95% CI –106 to –17, *P*=.08). The performance distribution of each group is shown in Figure 2.

Performance for the time variable differed significantly depending on the participants’ gender (*F*_1,106_=14.42, *P*<.001). This effect was caused by men outperforming women in the novice group (*t*_63_=–4.68, *P*<.001). No significant differences in performance for gender were found in the other groups.

**Figure 3:**
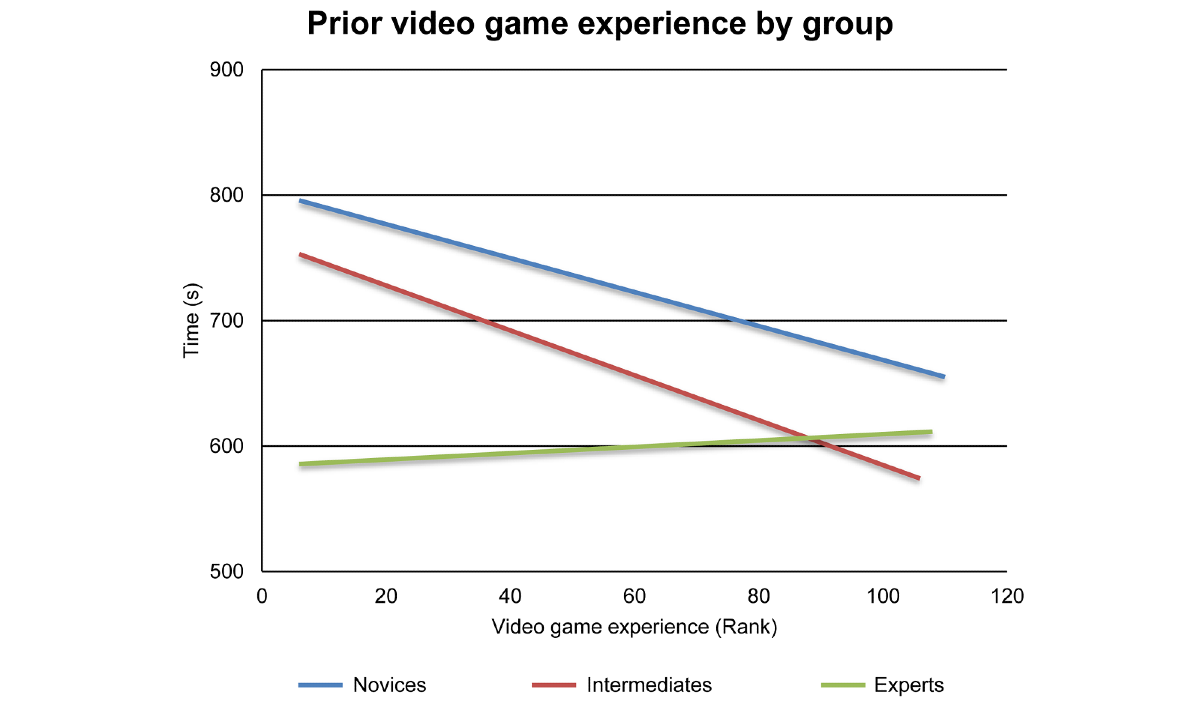
Linear trend lines showing the correlation between video game experience and the performance variable of time for each group.

Video game experience was also significantly related to the time performance variable (*F*_1,106_=5.20, *P*=.03). A correlation between video game experience and game completion time was observed for both novices and intermediates but not for experts. The linear trend lines for the video game experience of each group are shown in Figure 3.

We were not able to establish independence of the gender and prior video game experience variables.

### The Error Performance Variable

In all groups, the subjects made few errors, and data were only normally distributed within the intermediate group. The differences in error performance between the experience groups were qualitatively similar to the differences in time performance but did not reach statistical significance. A floor effect and a limited range of errors were observed, ranging from 0-6 (Table 2).

**Table 2:** Numbers of participants who made 0, 1-3, and 4-6 errors in each group.

Errors, n	Group, n (%)		
	Novice (n=65)	Intermediate (n=26)	Expert (n=20)
0	3 (5)	3 (12)	4 (20)
1-3	38 (58)	18 (69)	16 (80)
4-6	24 (37)	5 (19)	0 (0)

## Discussion

### Principal Findings

In this study, we demonstrated construct validity for the laparoscopic serious game *Underground*. The completion times for the investigated game levels differed between 2 pairs of the 3 paired groups; the novices were outperformed by both other groups, but there was no difference between the intermediates and experts. The lack of performance difference between the intermediate and expert groups is likely because *Underground* was developed to provide *basic* laparoscopic skills training, a skill level that is already mastered by intermediates and experts. After previous studies established face validity, concurrent validity, and partial construct validity, we now provide additional construct validity [16-18]. *Underground* is a welcome addition to the existing laparoscopic simulation landscape.

Measurement of the error variable was not sufficiently sensitive for a full statistical analysis. Since *Underground* was not developed with the intent to provide formal laparoscopic performance assessments, we created custom time and error variables as described in the Methods section. Although our results for the error variable did not demonstrate significance, we have included them here because there were trends toward differences in error performance between the experience groups; also, we feel it is important that surgical performance assessment include variables beyond the speed of task completion, as speed in itself is not informative of the quality of the performance [6,29].

On its own, *Underground* has very limited built-in capabilities to measure performance. This lack of reliable performance metrics is one of its biggest limitations at present [20,24]. In the course of conducting our pilot study, it became clear that the game-supplied variables (number of robots saved and number of bonus items collected) did not discriminate between laparoscopic experience, as nearly all pilot study participants achieved the maximum score regardless of laparoscopic experience. Well-developed in-game performance metrics would improve the usefulness of *Underground* for basic laparoscopic skills training.

Future developments in the area of serious gaming for laparoscopic skills development would benefit from a more formal approach to the development of in-game metrics. Delphi method-based rounds that include students, experts from the video game industry, content experts (eg, surgeons), and educational psychologists may help unlock the full potential of serious gaming by combining insights from each of these complementary professional groups. With regard to *Underground* specifically, continued support of its developers could overcome the current lack of informative parameters in the game by providing a game update via a patch, as is commonplace for contemporary video games. The low cost and small form factor of *Underground* facilitate its installation in non-skills lab settings such as residents’ homes or offices. This flexibility may increase residents’ training volumes.

Given that *Underground* is a game-based educational tool, and given that gaming has increasingly become an integral part of residents’ and surgeons’ daily lives [30], we additionally assessed the effect of the subjects’ prior video game experience on their *Underground* gaming performance. A greater amount of prior gaming experience was found to be associated with faster performance in the novice and intermediate groups. These results are consistent with previous studies, where video game experience has been shown to improve baseline performance on simulators [31,32]. Interestingly, prior video game experience did not result in better *Underground* performance within the expert group. This finding can be explained by a generational difference in the subject groups, since the novices and intermediates generally had more video game experience than the experts. Alternatively, it is possible that experts’ video game experience made a negligible contribution to their performance compared with the impact of their professional experience. The literature is divided as to whether video game experience positively impacts operating room performance, with some studies finding a positive effect and others finding no impact [30,33-38].

Adopting *Underground* in our training curricula corresponds well with the current interest in multimodality training, which in the context of surgical training refers to the use of different simulations to train specific surgical skills [39]. Several studies have shown that junior residents particularly benefit from multimodality training for mastering basic laparoscopic skills [39-41]. This training provides the trainee with a fresh perspective each time a new modality is used, which enhances their learning [39]. It would be beneficial to residents to broaden this approach even further by including games such as Touch Surgery for training the procedural aspects of surgical procedures in addition to basic psychomotor skills [42-45].

### Limitations

Our experience groups comprised different gender ratios, reflecting the increase in the number of women enrolling in medical school. As a result, our novice group had a greater proportion of female participants than the intermediate and expert groups. However, gender only impacted the results in the novice group. Since female participants in this group also showed a difference in gaming experience, we were not able to independently assess the effect of gender. Given the lack of impact of gender on the other 2 groups, we think the gender effects in the novice group are caused by differences in gaming experience rather than gender differences per se.

Methods for validity testing are changing. Currently, 2 main frameworks are used in the literature: the classic validity framework, which includes validity sources such as face, content, construct and concurrent validity, and the modern framework proposed by Messick et al [46], which is a unified model in which different sources of validity are explored. In our studies of serious gaming, we opted to use the older framework so that our results would be comparable with those of other literature reports in this area (eg, Jalink et al [18]). This provides a better benchmark regarding the potential of *Underground* as a serious game for laparoscopic training. In the more current Messick framework, our study would support “relations to other variables” as sources of validity [46,47].

### Future Research

Understanding which specific aspects of the serious game *Underground* are responsible for laparoscopic skills transfer would aid its adoption in the surgical skills curriculum [48,49]. It is possible that the specialized laparoscopic gaming interface, compared to the original Wii U GamePad, is a critical component influencing laparoscopic skills transfer; this effect should be investigated further. In addition, if skill transfer to the operating room can be established, the potential value of serious games such as *Underground* will be heightened both for future laparoscopic skills training and for use as a preoperative warmup tool [50].

### Conclusion

This study establishes construct validity for the laparoscopic serious game *Underground*. Serious gaming may aid laparoscopic skill development. Gaming experience had an independent, positive effect on *Underground* performance compared to laparoscopic experience. The in-game performance metrics were not suitable for statistical evaluation. To unlock the full potential of serious gaming for training, a more formal approach to the development of performance metrics is needed.

## Abbreviations

ANCOVA: analysis of covariance
